# Ultrastable and Low-Threshold Two-Photon-Pumped Amplified Spontaneous Emission from CsPbBr_3_/Ag Hybrid Microcavity

**DOI:** 10.3390/nano14201622

**Published:** 2024-10-10

**Authors:** Shulei Li, Yatao Zhang, Zhiran Zhao, Shiyi Cheng, Zixin Li, Yuanyuan Liu, Quantong Deng, Jun Dai, Yunbao Zheng, Zhenxu Lin

**Affiliations:** 1 School of Optoelectronic Engineering, Guangdong Polytechnic Normal University, Guangzhou 510665, China; shuleili@gpnu.edu.cn (S.L.); ytaozhang0918@163.com (Y.Z.); zhiranzhao8@163.com (Z.Z.); shiyicheng18732@163.com (S.C.); zixinli2024@163.com (Z.L.); yuanyuanliu2024215@163.com (Y.L.); dengqt215@163.com (Q.D.); yunbaozheng@gpnu.edu.cn (Y.Z.); 2 School of Material Science and Engineering, Hanshan Normal University, Chaozhou 521041, China

**Keywords:** perovskite, superstructure, hybrid microcavity, thermal accumulation, amplified stimulated emission

## Abstract

Halide perovskite materials have garnered significant research attention due to their remarkable performance in both photoharvesting photovoltaics and photoemission applications. Recently, self-assembled CsPbBr_3_ superstructures (SSs) have been demonstrated to be promising lasing materials. In this study, we report the ultrastable two-photon-pumped amplified stimulated emission from a CsPbBr_3_ SS/Ag hybrid microcavity with a low threshold of 0.8 mJ/cm^2^ at room temperature. The experimental results combined with numerical simulations show that the CsPbBr_3_ SS exhibits a significant enhancement in the electromagnetic properties in the hybrid microcavity on Ag film, leading to the uniform spatial temperature distribution under the irradiation of a pulsed laser, which is conducive to facilitate the recrystallization process of the QDs and improve their structural integrity and optical properties. This study provides a new idea for the application of CsPbBr_3_/Ag hybrid microcavity in photonic devices, demonstrating its potential in efficient optical amplification and upconversion lasers.

## 1. Introduction

Achieving visible lasers by means of infrared two/multiphoton pumping has been demonstrated as an effective method for frequency upconversion [1,2,3]. Compared to single-photon excitations, double/multi-photon excitation has obvious advantages such as large penetration depth, small Rayleigh scattering, and small light damage to samples [4,5,6]. However, such lasers impose stringent requirements on large optical gain and efficient multiphoton absorption [7]. This restricts the range of lasing materials to a limited selection of organic dyes [8], polymers [9], and specific inorganic semiconductor nanostructures [10]. Among the suitable systems, metal halide perovskite (CsPbX_3_, (X = Cl, Br and I)) has attracted extensive attention and in-depth research [11,12,13]. This new kind of semiconductor is favored for its unique advantages in the field of optoelectronics, such as its high optical absorption coefficient, tunable band gap, long carrier lifetime, and mobility, which make it show great potential in photovoltaic, light-emitting diode (LED) [14], light detector, and laser applications [15,16]. However, their usage is significantly limited by their poor stability when exposed to moisture. Very promising approaches for overcoming these limitations have been discovered in many ways, among which encapsulation in inorganic oxidized glass is a more common approach [17,18]. To date, stable and low-threshold amplified stimulated emission (ASE) has been demonstrated from CsPbX_3_.

As the shape, size, and heterostructures of conventional CsPbX_3_ are further modified, the two-photon absorption in perovskite nanomaterials may be further enhanced [19,20,21]. The self-assembly and regrowth of individual components is one of the most facile approaches [22,23,24,25]. The CsPbX_3_ superstructure (SS) is formed through the self-assembly of QDs driven by weak microscopic interactions, resulting in a long-range-ordered three-dimensional structure [26]. In the process of the self-assembly of QDs, lattice and surface defects are generated due to factors such as interface mismatch, stress concentration, or unstable growth conditions [7,27]. These defects will cause an increase in the non-radiative recombination rate and a decrease in quantum efficiency. In addition, the low efficiency of the two-photon absorption process, which requires a high-power excitation source, produces significant thermal effects, resulting in the thermal damage or photodegradation of the material. Therefore, the performance of two-photon-pumped ASE largely depends on the crystallinity and morphology of the CsPbX_3_ structure. Laser annealing is the use of laser energy to achieve the local or overall heating of the material [28,29,30,31], which can effectively repair various defective materials, so as to significantly improve the optical properties and stability of the material [32,33,34]. However, the thermal management in a CsPbX_3_ SS during laser annealing is necessary to avoid thermal accumulation on the surface of the CsPbX_3_ SSs. The application of optical microcavities shows potential in enhancing thermal control during this process [35,36,37,38,39]. It is reported that optical microcavities enhance temperature distributions, and reduce thermal damage by limiting and localizing electromagnetic fields [33,40]. It is reported that optical microcavities enhance temperature distributions, and reduce thermal damage by limiting and localizing electromagnetic fields. In addition, the resonance mode in the microcavity can improve the efficiency of energy transfer.

Herein, we formed CsPbBr_3_ SSs through the self-assembly of QDs, combined with silver film to prepare a CsPbBr_3_/Ag hybrid microcavity. Femtosecond laser annealing technology was used to eliminate SS surface defects and lattice defects. The hybrid microcavity has a plasma–photon hybrid mode, the electromagnetic environment inside the SS is redesigned, and the temperature distribution during the laser annealing process is precisely controlled to facilitate the recrystallization process of the QDs, improving their structural integrity and optical properties. The laser threshold is lowered, which achieves an efficient ASE and upconversion emission. This is of great significance for the realization of high-efficiency and low-threshold amplified spontaneous radiation and two-photon-pumped laser.

## 2. Materials and Methods

### 2.1. Sample Preparation and Characterization

In this work, the CsPbBr_3_/Ag hybrid microcavities used was fabricated by the following procedure. First, CsPbBr_3_ QDs were synthesized by hot-injection method. The as-prepared CsPbBr_3_ QD solution with the added methyl acetate was left standing at room temperature for ten days. Then, the solution was spin-coated onto a Ag/SiO_2_ substrate and SiO_2_ substrate at a rate of 4000 rpm for 40 s. The CsPbBr_3_ SSs were formed on the surface of the Ag film and SiO_2_ substrate, respectively. The morphology and component elements of CsPbBr_3_ SSs were characterized by a scanning electron microscope (SEM) (Hitachi SU5000, Tokyo, Japan) and energy dispersive spectroscopy (EDS) (Bruker EDS QUANTAX, Billerica, MA, USA). The PL spectra of CsPbBr_3_ SSs were collected by a 40× UV objective lens in a Raman spectrometer system (Horiba LabRAM HR Evolution, Tokyo, Japan) equipped with a 325 nm He-Cd laser (Kimmon, Tokyo, Japan).

### 2.2. Optical Characterization of CsPbBr_3_/Ag Hybrid Microcavities

The 800 nm femtosecond laser (Mira 900S, Coherent, Saxonburg, PA, USA, 130 fs) was utilized to excite the CsPbBr_3_ microdisks (SSs). The PL spectra measurements were conducted at a high repetition rate of 76 MHz, and luminescence lifetimes were measured using a low repetition rate of 3.8 MHz (achieved via a pulse picker) in conjunction with a time-correlated single-photon counting system (lifespec II, Edinburgh Instruments, Livingston, UK). The laser light was focused onto the samples using a 50× objective lens (NA = 0.8) on an inverted microscope (Axio Observer A1, Zeiss, Oberkochen, Germany). The scattered light and photoluminescence were collected through the same objective lens and directed either to a spectrometer (SR-500i-B1, Andor, Abingdon, Oxon, UK) for spectral analysis or to a charge-coupled device (DU970N, Andor, Abingdon, Oxon, UK) for recording.

### 2.3. Numerical Simulation and Analytical Model

The electric field distribution of CsPbBr_3_ SSs placed on a thin Ag film and SiO_2_ substrate were numerically calculated using the finite-difference time-domain (FDTD) method (FDTD solution, https://www.lumerical.com (accessed on 1 August 2024)). The side length of the CsPbBr_3_ SSs is 1.5 × 1.5 μm and the height is 0.5 μm. The dielectric constants of Ag and CsPbBr_3_ SSs were taken from the previous literature [41,42]. The refractive index and thermal conductivity of CsPbBr_3_ SSs were taken from the literature. The temperature distributions inside the hybrid microcavities were calculated numerically based on the finite element method (FEM) (COMSOL Multiphysics v5.6, https://www.comsol.com (accessed on 1 August 2024)). The complex refractive index and thermal conductivity of monocrystalline CsPbBr_3_ were taken from the literature [12,43,44].

## 3. Results and Discussion

**Figure 1:**
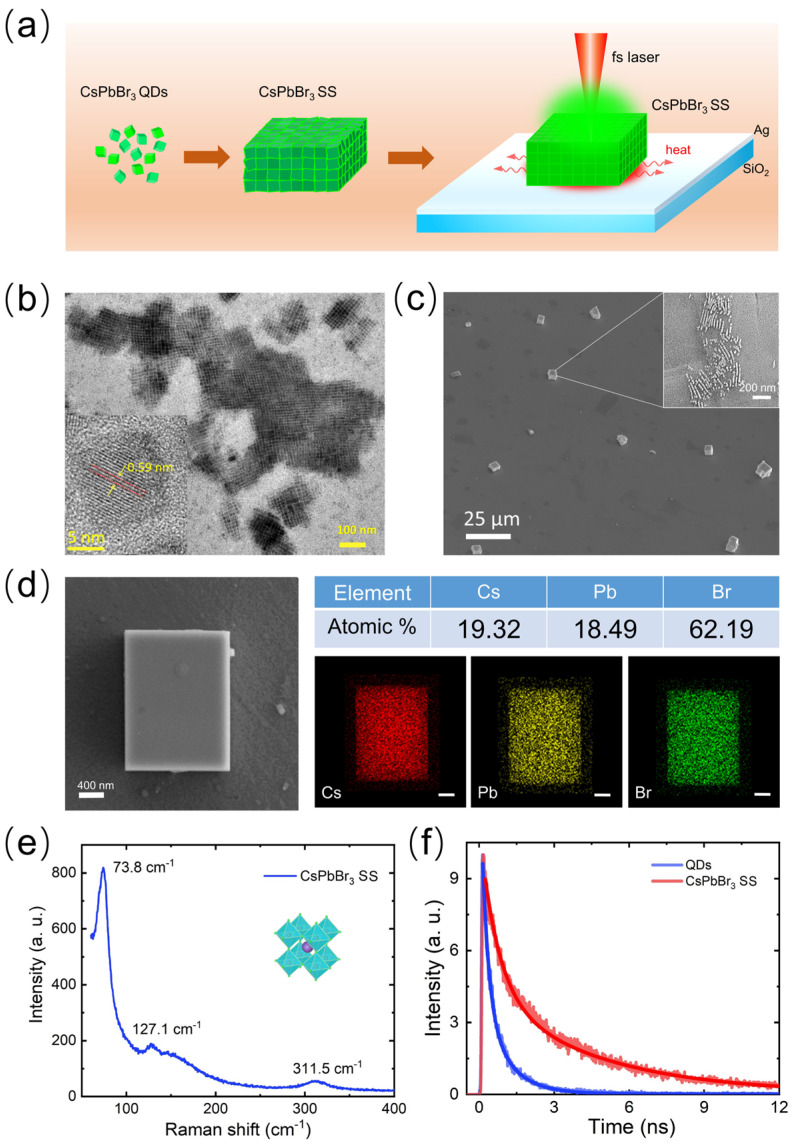
(**a**) Schematic of the CsPbBr_3_ SS formation process and repairing internal defects of CsPbBr_3_ SS by laser annealing. (**b**) TEM image of monodispersed CsPbBr_3_ QDs. The inset shows a high-resolution image. (**c**) The SEM image of CsPbBr_3_ SSs on a Ag film surface. The inset shows QDs remaining on the top of SSs. (**d**) SEM image of a CsPbBr_3_ SS on the Ag/SiO_2_ substrate, EDS analysis of Cs, Pb, Cl, Br. Scale bar: 400 nm. (**e**) Raman spectrum measured for a CsPbBr_3_ SS. The crystal structure of CsPbBr_3_ SS is shown in the inset. (**f**) PL decays measured for CsPbBr_3_ QDs and CsPbBr_3_ SS placed on the Ag/SiO_2_ substrate. In both cases, the PL decays are fitted by bi-exponential decay functions with two-time constants.

Figure 1a shows a schematic diagram of the CsPbBr_3_/Ag hybrid microcavity prepared using interfacial self-assembly, spin-coating, and post-self-assembly annealing. First, a polar solvent (acetone) is added to the CsPbBr_3_ QDs solution to remove the oleic acid/oleylamine ligands on the surface of QDs, thereby enhancing the weak interactions between them. This process allowed the QDs to slowly self-assemble, forming an orderly arranged microscale superstructure, known as CsPbBr_3_ SSs. Then, a CsPbBr_3_/Ag hybrid microcavity was prepared by placing a CsPbBr_3_ SS on the surface of the Ag film. In a previous study, the coupling between the exciton emissions of the CsPbBr_3_ QDs and microsphere WGM optical mode on Ag film substrate was significantly stronger than that on Au film substrate and glass substrate [16]. This is due to the lower optical loss of the silver film, and the plasmon resonance wavelength is closer to the ultraviolet region. The hybrid nanocavity structure composed of dielectric particles and metal film can form a plasma–photon mixed mode, thereby redesigning the electromagnetic environment inside the dielectric particles. The self-assembly of QDs can lead to the formation of defects, such as surface defects, lattice defects, and uneven QD coupling, which can be repaired under laser annealing. The ordered structure of CsPbBr_3_ SS is formed on the microscale, thus improving the crystal quality and structural integrity.

The CsPbBr_3_ SSs were fabricated by the self-assembly of QDs, assisted by polar solvent. Figure 1b shows the high-resolution transmission electron microscope (HR-TEM) image of pristine monodispersed CsPbBr_3_ QDs with an average size of ~10 nm. The HR-TEM images reveals distinct lattice fringes with an interplanar spacing of ~0.59 nm. The self-assembly of QDs is primarily driven by intermolecular forces between their aliphatic ligands. In this study, surface-bound ligands, which are highly responsive to polar solvents, were utilized to assemble CsPbBr_3_ SSs. Figure 1c shows that the CsPbBr_3_ SSs appear as cuboids with micrometer edges. There are still some QDs remaining on the surface of SSs that are not involved in synthesis, but they also show regular arrangement. In Figure 1d, the SEM image reveals that the CsPbBr_3_ SSs have a smooth and flat surface, featuring a microscale superstructure with an orderly arrangement. The elemental mapping of a typical CsPbBr_3_ SS based on energy-dispersive spectroscopy (EDS) is presented. The atomic ratio of Cs–Pb–Br is very close to the stoichiometry of 1:1:3. In Figure 1e, we present the Raman spectrum of the CsPbBr_3_ SSs. As reported previously, a strong peak at 73.8 cm^−1^ corresponds to the acoustic phonon mode, which is attributed to the vibration mode of the [PbBr_6_]^4−^ octahedron. Additionally, a broad peak at 127.1 cm^−1^ is associated with the motion of Cs^+^ cations, and the broad peak at 311.5 cm^−1^ is related to the second-order phonon mode of the octahedron [45]. We also measured the luminescence lifetimes of CsPbBr_3_ QDs and CsPbBr_3_ SSs, as shown in Figure 1f. The PL decay curves were fitted using a bi-exponential decay function with two-time constants (τ_1_ and τ_2_). The results indicate that the luminescence lifetime of the CsPbBr_3_ SSs (τ_1_ = 2.3 ns, τ_2_ = 4.5 ns) is significantly longer than that of the QDs (τ_1_ = 0.45 ns, τ_2_ = 0.93 ns). This extended luminescence lifetime is due to the suppression of nonradiative recombination, as the interactions between QDs reduce nonradiative pathways and enhance radiative recombination. Moreover, the CsPbBr_3_ SS induces photon localization effects, which decrease the energy exchange efficiency between the QDs and their surrounding environment, leading to prolonged fluorescence lifetimes. A comparison of the PL peaks between the QDs and the microdisks reveals a redshift in the peak position. This redshift can be attributed to electronic coupling within the superlattice, which causes the splitting of the quantized carrier energy levels of individual QDs and the formation of quasi-collective electronic bands.

**Figure 2:**
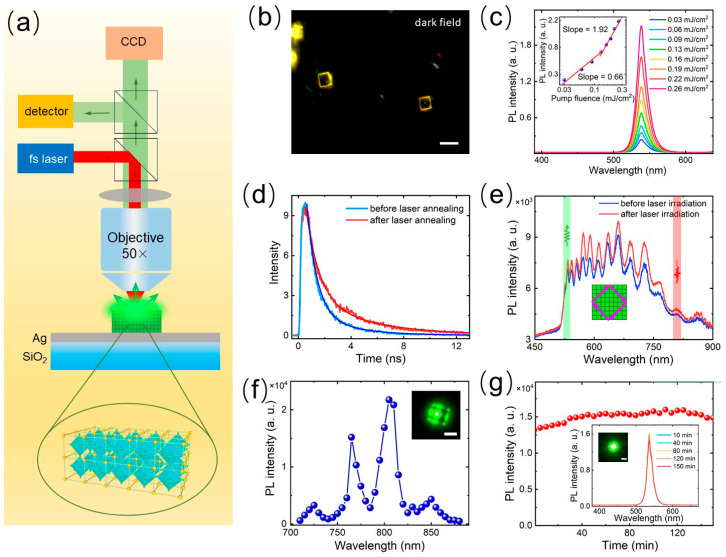
(**a**) Schematic showing the experimental setup used to investigate the optical properties of a CsPbBr_3_/Ag hybrid microcavity. (**b**) Optical image of CsPbBr_3_ SS placed on the Ag/SiO_2_ substrate. Scale bar: 4 μm. (**c**) PL spectra measured for a CsPbBr_3_ SS placed on the Ag/SiO_2_ substrate excited using 800 nm femtosecond laser pulses of 76 MHz at different laser powers. The inset shows the dependence of the PL intensity on the pump fluence. (**d**) PL decays measured for a CsPbBr_3_ SS placed on the Ag/SiO_2_ substrate before and after laser annealing. (**e**) Scattering spectra measured for a CsPbBr_3_ SS placed on the Ag/SiO_2_ substrate before and after the pump fluence. (**f**) PL spectra measured for a CsPbBr_3_ SS placed on the Ag film at different pumping wavelengths in the range of 720–880 nm. Scale bar: 2 μm. (**g**) Integrated PL intensities of the CsPbBr_3_/Ag hybrid microcavity under the pump fluence using 800 nm femtosecond laser pulses of 76 MHz at different times. The inset shows the emission spectra of the CsPbBr_3_/Ag hybrid microcavity measured at different times. Scale bar: 2 μm.

Laser annealing has been widely used to repair the defects of micro and nano materials. It uses high-energy laser pulses to heat the surface of the material instantaneously, prompting the local temperature to rise rapidly so that the atoms in the lattice are rearranged and thus repair or reduce the defects in the material. The experimental setup used in this work is illustrated in Figure 2a [33,46,47,48]. The backscattering and PL of CsPbBr_3_ SSs were collected with a microscope, analyzed with a spectrometer, and images were collected with CCD. Figure 2b shows the dark field image of a CsPbBr_3_ SS placed on the surface of Ag film. The CsPbBr_3_ SS was annealed by using 800 nm femtosecond laser pulsed with repetition rate of 76 MHz at a power fluence of 0.2 mJ/cm^2^. In this case, using conventional point excitation, the CsPbBr_3_ SS was irradiated for 1 min. Figure 2c shows the upconversion PL spectrum of CsPbBr_3_ SS under different power. To confirm the two-photon absorption (TPA) and emission process of CsPbBr_3_ SSs, we show the dependence of PL intensity on laser power, noting that the slope of intensity increase was significantly lower at low laser power compared to high laser power. This behavior is mainly due to the significant changes between the QDs within the CsPbBr_3_ SSs, including TPA, field enhancement, and emission quantum efficiency. In this case, part of the energy absorbed is used to repair the internal defects of CsPbBr_3_ SSs, thus forming an atomic-scale-ordered structure in the microscale structure. After laser annealing, the quadratic relationship between PL intensity and excitation intensity clearly confirms the two-photon absorption and emission process. In Figure 2d, we show the luminescence lifetimes measured for CsPbBr_3_ SSs before and after laser annealing. It was found that the luminescence lifetime of CsPbBr_3_ SSs after laser annealing (τ_1_ = 1.5 ns, τ_2_ = 4.7 ns) is significantly longer than that before laser annealing (τ_1_ = 0.9 ns, τ_2_ = 3.3 ns). Additionally, we were surprised to observe an increase in scattering intensity and a blue shift in the peak position after annealing, as shown in Figure 2e. This indicates that some voids and vacancies still exist within the CsPbBr_3_ SSs, and the localized heating caused by the laser annealing rearranges or passivates uncoordinated ions on the QDs surfaces, reducing the number of surface defect states and promoting the recrystallization process. Then, a CsPbBr_3_ SS/Ag hybrid nanocavity has good crystallization quality after laser annealing and can achieve high-stability fluorescence emission. In Figure 2f, we further investigated the dependence of PL intensity on an excitation wavelength, finding that the maximum efficiency was achieved at 800 nm, which coincides with the optical resonance peak of the CsPbBr_3_/Ag hybrid microcavity. This resonance-enhancement effect increases the absorption efficiency of the CsPbBr_3_ SS at specific wavelengths, which is crucial for achieving low-threshold spontaneous emission. We further studied the stability of fluorescence emission. Figure 2g shows that, when a 800 nm, 76 MHz pulsed laser is irradiated with 1.1 mJ/cm^2^ for about 2.5 h, the light intensity of CsPbBr_3_ SS remains stable and has good light stability.

**Figure 3:**
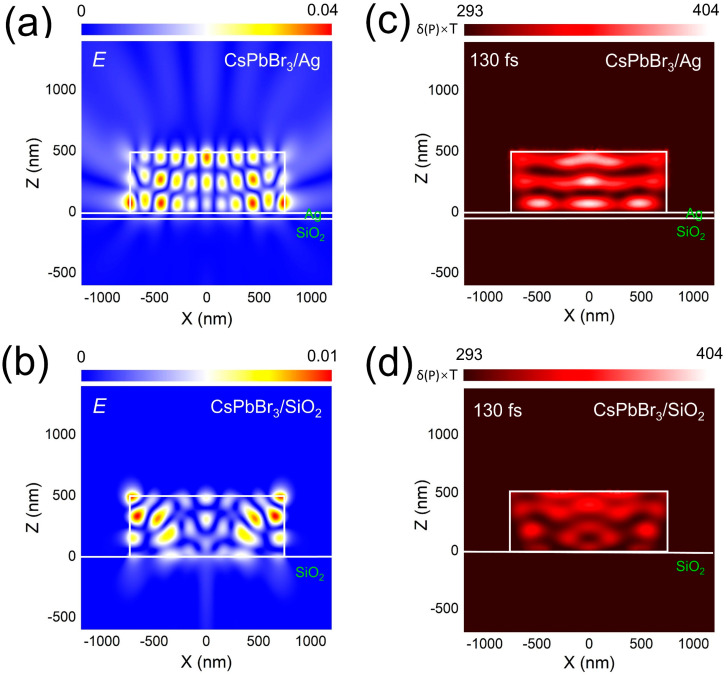
(**a**) Electric field distributions calculated for the CsPbBr_3_ SS placed on the Ag/SiO_2_ substrate at wavelengths of 540 nm. (**b**) Electric field distributions calculated for the CsPbBr_3_ SS placed on the SiO_2_ substrate at wavelengths of 540 nm. (**c**) Transient temperature distribution in the XZ planes (t = 130 fs) calculated for a CsPbBr_3_ SS placed on the Ag/SiO_2_ substrate and excited by using a single 800 nm femtosecond laser pulse with a duration of 130 fs. (**d**) Transient temperature distribution in the XZ planes (t = 130 fs) calculated for a CsPbBr_3_ SS placed on the SiO_2_ substrate and excited using a single 800 nm femtosecond laser pulse with a duration of 130 fs.

It is well known that the electric field has an important effect on temperature distribution, especially at the micron- and nanoscale. The electric field has a profound effect on the regulation of local temperature, which is a very interesting and complex problem. Until now, however, accurately measuring the temperature inside a micron cavity has been a challenge. Therefore, we simulated the transient temperature distribution within CsPbBr_3_ SSs on different substrates under pulsed femtosecond laser irradiation, using a femtosecond laser two-temperature model [49]. In Figure 3a, we present the XZ-plane electric field distribution of a CsPbBr_3_ microdisk (SS) placed on a Ag thin film at a wavelength of λ = 540 nm, which corresponds to the emission wavelength of CsPbBr_3_. Here, the side length and thickness of the cubic SS are assumed to be 1.5 μm and 0.5 μm, respectively. Notably, the electric field distribution within the CsPbBr_3_ SS exhibits standing wave patterns in both the x and z directions. This regular electric field distribution arises from the interaction between higher-order Mie resonances and the whispering gallery mode (WGM) or Fabry–Pérot (F–P) resonances supported by the hybrid microcavity [16]. The electric field distribution within a CsPbBr_3_ SS on a glass substrate, as shown in Figure 3b, is irregular, with indistinct resonance modes and a weaker field strength. It is found that the low radiation loss of the CsPbBr_3_/Ag hybrid optical mode is the reason for the high-efficiency fluorescence emission of the CsPbBr_3_ SS, which is conducive to the light amplification of the CsPbBr_3_/Ag hybrid microcavity. Figure 3c shows the temperature distribution of CsPbBr_3_ SS placed on the surface of an Ag film under single-femtosecond laser pulse excitation. The contact area between the CsPbBr_3_ SS and Ag film has an obvious spatial region, and the temperature distribution is layered under the joint action of the internal WGM and F-P cavity, and the spatial temperature distribution is more uniform, which is conducive to the repair of internal defects. We also calculated the temperature distribution of CsPbBr_3_ SS on the glass substrate. The temperature distribution was mainly concentrated on top, and the temperature distribution was uneven and significantly lower than that of CsPbBr_3_ SS on the surface of the Ag film, as shown in Figure 3d.

**Figure 4:**
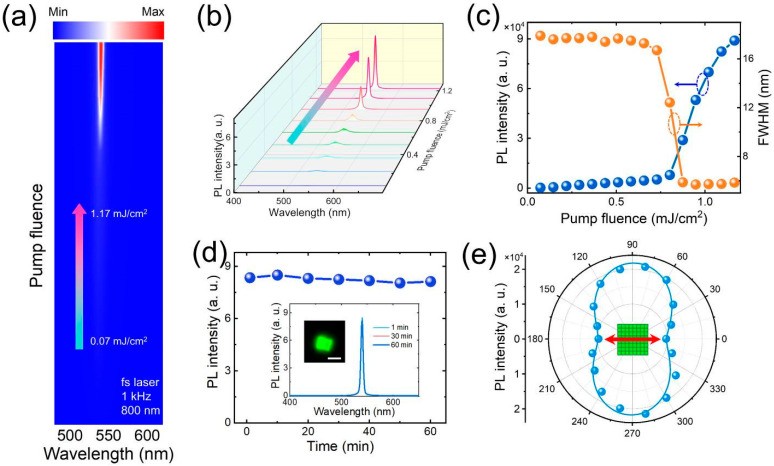
(**a**) The pump-fluence-dependent PL emission from the CsPbBr_3_/Ag hybrid microcavity obtained using 800 nm femtosecond laser pulses of 1 kHz had a pump intensity range of 0.07–1.17 mJ/cm^2^. (**b**) The pump-fluence-dependent PL emission spectra of CsPbBr_3_/Ag hybrid microcavity obtained using 800 nm femtosecond laser pulses of 1 kHz. (**c**) The variation tendency of the PL intensity and FWHM of CsPbBr_3_/Ag hybrid microcavity with increasing pump fluence. (**d**) Integrated PL intensities of the CsPbBr_3_/Ag hybrid microcavity under the pump fluence using 800 nm femtosecond laser pulses of 1 kHz at different times. The inset shows the emission spectra of the CsPbBr_3_/Ag hybrid microcavity measured at different times. Scale bar: 2 μm. (**e**) Dependence of the PL intensity on the polarization angle obtained for the CsPbBr_3_/Ag hybrid microcavity. The polarization of the laser light is marked by red arrow.

High threshold and instability have been major drawbacks limiting the applications of two-photon-pumped ASE and lasers. We further explore the ASE induced by the two-photon pumping of CsPbBr_3_ SS. The stimulated emission of the CsPbBr_3_/Ag hybrid microcavity was measured at room temperature using a femtosecond laser pulse of 800 nm and 1 kHz. Figure 4a,b shows the PL emission of CsPbBr_3_ SS at different pump intensities. It is found that, at a relatively low excitation intensity (<~0.8 mJ/cm^2^), the PL spectrum is determined by spontaneous radiation with a half-peak full width (FWHM) of ~20 nm. With the further increase in pump intensity, a new ASE narrow peak with a line width of about ~5 nm appears, indicating that two-photon absorption achieves the frequency upconversion-stimulated emission, as shown in Figure 4c. The spectral integral PL intensity on the narrow peak increases abruptly from a certain point with the pumping intensity, which further indicates that CsPbBr_3_ nanocrystals have two-photon-pumped stimulated emission. The threshold of ASE produced by the CsPbBr_3_ SS/Ag hybrid microcavity reaches 0.8 mJ/cm^2^, which is lower than that of the reported CsPbBr_3_ films or microchips. This low threshold is due to the enhanced light field intensity in the optical microcavity, where multiple optical resonances and well-distributed electric fields improve light confinement and amplification, reducing radiation losses and increasing emission efficiency. This enhancement effect reduces the laser threshold, allowing ASE to be achieved with a lower input excitation power. An important property of the optical gain material is its optical stability under the irradiation of the pumped laser. In order to obtain the optical stability of CsPbBr_3_ SS by two-photon pumping, the peak intensity of stimulated emission was monitored at 800 nm as a function of the pumped laser pulse. As shown in Figure 4d, the stimulated peak intensity can easily last for 1 h of laser irradiation. Figure 4e shows the polarization characteristics of PL fluorescence. These results indicate that the CsPbBr_3_/Ag hybrid microcavity is an ideal material for gain media in high-performance upconversion lasers.

## 4. Conclusions

In summary, we report a strategy to achieve stable low-threshold ASE under two-photon excitation in CsPbBr_3_/Ag hybrid microcavities through self-assembly and laser annealing techniques at room temperature. The results demonstrate that the self-assembled CsPbBr_3_ SS exhibits significantly enhanced electromagnetic properties in the hybrid microcavity on the Ag film, thus improving the interaction efficiency between light and matter and leading to the uniform spatial temperature distribution under the irradiation of a pulsed laser. As a result, the defects, generated during the QD self-assembly process, can be effectively repaired in such a hybrid microcavity. It is conducive to enhance the crystal quality and optical performance of the SS. Experimental characterization and simulation analysis indicate that this hybrid microcavity structure possesses excellent photostability and low-threshold stimulated emission characteristics, suggesting its potential application in two-photon-pumped lasers. Our findings indicate the important role of CsPbBr_3_/Ag-hybrid microcavity in enhancing the performance of photonic devices and provide a valuable theoretical and experimental reference for the development of new photonic materials.

## Data Availability

The data presented in this study are available upon request from the corresponding author.
